# A Comprehensive Review of the Therapeutic Value of Urine-Derived Stem Cells

**DOI:** 10.3389/fgene.2021.781597

**Published:** 2022-01-03

**Authors:** Qian Zhou, Yiyu Cheng, Fang Sun, Jie Shen, M. I. Nasser, Ping Zhu, Xueyan Zhang, Yuxiang Li, Guangming Yin, Yuequn Wang, Xiushan Wu, Mingyi Zhao

**Affiliations:** ^1^ Department of Pediatrics, The Third Xiangya Hospital, Central South University, Changsha, China; ^2^ Guangdong Cardiovascular Institute, Guangdong Provincial People’s Hospital, Guangdong Academy of Medical Sciences, Guangzhou, China; ^3^ Department of Urology, The Third Xiangya Hospital, Central South University, Changsha, China; ^4^ The Center for Heart Development, State Key Laboratory of Development Biology of Freshwater Fish, Key Laboratory of MOE for Development Biology and Protein Chemistry, College of Life Sciences, Hunan Normal University, Changsha, China

**Keywords:** urine-derived stem cells, multiple differentiation, molecular mechanisms, disease treatment, drug discovery, tissue engineering

## Abstract

Stem cells possess regenerative powers and multidirectional differentiation potential and play an important role in disease treatment and basic medical research. Urine-derived stem cells (USCs) represent a newly discovered type of stem cell with biological characteristics similar to those of mesenchymal stromal cells (MSCs), including their doubling time and immunophenotype. USCs are noninvasive and can be readily obtained from voided urine and steadily cultured. Based on advances in this field, USCs and their secretions have increasingly emerged as ideal sources. USCs may play regulatory roles in the cellular immune system, oxidative stress, revascularization, apoptosis and autophagy. This review summarizes the applications of USCs in tissue regeneration and various disease treatments. Furthermore, by analysing their limitations, we anticipate the development of more feasible therapeutic strategies to promote USC-based individualized treatment.

## Introduction

Stem cells, including embryonic stem cells (ESCs), adult stem cells (ASCs) and induced pluripotent stem cells (iPSCs), have increasingly emerged as research hot spots due to their roles in sustaining tissue homeostasis and regeneration and promoting tissue repair and responses (Daley, 2015; Dayem et al., 2020). Although significant progress has been made in stem cell research, ESC applications are still limited by ethical and tumour formation issues. Nevertheless, ASCs have gradually attracted increasing attention because they have expanded our understanding of disease pathogeneses. Multiple types of ASCs have been identified, such as mesenchymal stem cells (MSCs) and haematopoietic stem cells (HSCs). Among them, MSCs are an important type of ASC that can be separated from human bone marrow (Baker et al., 2015), adipose tissue (Mazini et al., 2020) and urine (Liu et al., 2021). Bone marrow-derived mesenchymal stem cells (BMSCs), adipose tissue-derived stem cells (ADSCs) and urine-derived stem cells (USCs), all of which have been demonstrated to be capable of self-renewal and multidirectional differentiation, regenerate corresponding tissues, such as functional bone, muscle, nerve, and endothelial tissue (Zhang et al., 2014). Among ADSCs, BMSCs and USCs, USCs have been noted to exhibit a higher proliferative rate and vascularization potential and to have better adipogenic, myogenic, neurogenic and endothelial differentiation potential with high efficiency; moreover, the collection of USCs is advantageously noninvasive and inexpensive (Kang et al., 2015; Wu et al., 2018). Therefore, the study of USCs could be of great value.

USCs were first identified by Zhang et al. (2008), and their autologous application represents a novel breakthrough in stem cell research. USCs exhibit a strong proliferation ability and are capable of multidirectional differentiation from MSCs isolated from urine. USCs can be isolated directly from urine by centrifugation and inoculated into culture medium, which is a simple, inexpensive and noninvasive procedure, while the overwhelming majority of ASCs (such as BMSCs and ADSCs) require invasive and painful collection procedures, which could be problematic for donors (Brown et al., 2019). In addition, a study found that a single cell can form 5–7 cell clones/100 ml urine at 2–3 days and then individually expanded to a large number of USCs due to their higher telomerase activity and long telomeres (Bharadwaj et al., 2013; Lang et al., 2013). USCs can be broadly applied to facilitate cutaneous regeneration and wound healing, attenuate osteoporosis, etc., (Chen et al., 2018a; Chen et al., 2019). Additionally, USCs have mesodermal differentiation potential and can differentiate into osteoblasts (OBs) or lipid-forming cells (Kim et al., 2020a). USCs have great potential and can produce donor-specific autologous cells for the repair of various tissues and the maintenance of organ functional integrity, thereby compensating for the shortcomings and deficiencies of existing treatment methods. For example, the current treatment options for patients with organ failure, such as drug therapies (Feng et al., 2020) and temporary replacement therapies (Dalal, 2015), are limited by objective factors. Especially in organ transplantation, in addition to the limited sources of organs, the subsequent need for long-term treatment with immunosuppressive agents to prevent immune rejection can have potentially fatal complications. Furthermore, autologous tissue transplantation is commonly utilized without immune rejection. In general, USCs have a wide range of potential applications in clinical cell therapy.

Similarly, most studies have shown that USCs exert therapeutic effects *via* paracrine mechanisms, which can be activated by extracellular vesicles (EVs), including exosomes and microvesicles. Jiang et al. first extracted exosomes from USCs after finding spherical vesicles that were approximately 50–100 nm in size (Jiang et al., 2016). Subsequently, Liu et al. found that these exosomes had the potential to facilitate angiogenesis and promote cell survival by producing growth factor (GF) and transforming growth factor-β1 (TGF-β1) (Liu and Wu, 2020). Studies have shown that, compared with USC implantation, USC-EV implantation has similar or even better therapeutic effects and that these cells possess the immune-privileged characteristics of their original cells. Moreover, USC-EVs are easier to store and manage and have a lower risk of tumour formation. USC-EV treatment is a superior strategy due to their efficacy, convenience and safety (Basu and Ludlow, 2016). Additionally, USC-derived induced pluripotent stem cells (USC-iPSCs) have recently become a hot research topic. These cells have been reprogrammed by retroviruses, mRNA transfection, and Sendai viruses, as exhibited by their high expression of the pluripotent genes SOX2, OCT4, NANOG, and CRIPTO/TDGF1 (Wang et al., 2017a; Cao et al., 2018; Kim et al., 2020b). Thus far, USC-iPSCs have been reported to rapidly proliferate and efficiently differentiate into many cell types with higher yields and less immunological rejection, mainly in endocrine systems (Guo et al., 2017a; Guo et al., 2017b) and digestive systems (Niemietz et al., 2016). In addition, USC-iPSCs can potentially be used to establish disease models based on their ability to provide an unlimited source of cells with the same genes as diseased cells (Fus-Kujawa et al., 2021). However, iPSCs are characterized by genetic instability and self-renewal ability, especially in regards to undifferentiated pluripotent stem cells forming tumours in the body, which is particularly worrying. In response to this problem, Ben-David et al. proposed the use of a small molecule targeting stearoyl-CoA desaturase 1. The molecule can selectively induce the death of undifferentiated iPSCs/ESCs and may be an effective therapeutic strategy with an improved safety profile. However, current research on the risk of using USC-iPSCs is limited, and these cells may become the focus of future research (Ben-David et al., 2013; Barreca et al., 2020). In the future, USC-iPSC-based treatment could potentially benefit humans.

Since the identification of their biological characteristics, USCs have been shown to be extracted easily and are regarded as an economical, convenient, and safe cell source with strong proliferation characteristics and *in vitro* multilineage differentiation potential. Here, we provide an overview of up-to-date studies investigating USCs and their therapeutic value in various diseases.

## Isolation and Culture of USCs

### Extraction of USCs

USCs were isolated by conventional centrifugation, and the impure cells were removed gradually. Tayhan et al. noted that air-isolated, cryopreserved urine from healthy young adults was the best sample for the extraction of fresh USCs. While some initial studies reported that USCs could not be extracted from diabetic patients (Tayhan et al., 2017), the latest research confirms that they can indeed be successfully extracted from these patients but that their regenerative ability is significantly reduced and they thus cannot be used for treatment (Xiong et al., 2019a). Therefore, appropriate sample selection is the premise for USC extraction. After the proper samples are collected, penicillin or streptomycin is added to reduce contamination. At room temperature, USCs can be isolated by centrifugation at 400 ×g for 10 min. Then, the supernatant is discarded, and the sample is washed twice with PBS and resuspended. Since urine contains different types of cells, it is necessary to identify the separated components after separation. The International Society for Cellular Therapy (ISCT) proposed that MSCs be identified by the following markers: 1) the cells can adhere to plastic culture bottles; 2) at least 95% of the cell population expresses specific surface antigens, including CD105, CD73 and CD90, and do not express CD45, CD34, CD14, CD11b, CD79α, CD19 or HLA-II antigens; and 3) the cells can be differentiated into OBs, chondroblasts and adipocytes under appropriate conditions (Dominici et al., 2006). Because USCs meet the criteria for MSCs, isolated USCs may express similar typical surface markers, such as CD29, CD44, CD54, CD73, CD90, CD166 and STRO-1 (Bharadwaj et al., 2013; Li et al., 2020; Xiang et al., 2020). In addition, pericyte (CD146) (Bharadwaj et al., 2013), epithelial (E-cadherin, claudin 1 and occludin), fibroblast (vimentin and fibronectin) and renal epithelial (L1CAM and NR3C2) (Kim et al., 2020b) markers are expressed in USCs.

### Culture of USCs

The most frequently used medium for USC culture is a 1:1 mixture of keratinocyte serum-free medium (KSFM, 50 μg/L basic fibroblast growth factor, 1% L-glutamine, 5 μg/L proepidermal growth factor, and 1% penicillin) and progenitor cell culture medium (75% DMEM, 25% F-12, 10% foetal bovine serum, 0.4 mg/L cortisol, 10 μg/L insulin, 5 μg/L transferrin, 2 × 10^−9^ mol/L triiodothyronine, 10 μg/L proepidermal growth factor, 1.8 × 10^−4^ mol/L adenine, 1% penicillin, and 1% L-glutamine). USCs are adherent and usually begin to form clones on the 5–7th day, reaching confluence on the 10th day (Lang et al., 2013; Burdeyron et al., 2020). A study reported the overall success rate of fresh urine cell cloning to be approximately 83% and the mean population doubling time of USCs to be 46–49 h (Lang et al., 2013). USCs are in high demand and in limited supply, and strategies such as better culture conditions and shorter culture periods *in vitro* can improve the challenges. For example, Chun et al. proposed the use of a reconstituted condition with an extracellular matrix (ECM) protein (collagen type I) and 5% O_2_ hypoxia as an optimized approach allowing for the expansion of harvested USCs while maintaining their chromosomal stability and multilineage differentiation potential (Chun et al., 2016). Gelatine, which is an ECM protein, was used as a coating material on a cell culture dish (Bharadwaj et al., 2013). A recent study reported that Matrigel, flavonoids and Y-27632 (a rho-associated protein kinase (ROCK) inhibitor) could be utilized to promote the efficiency of the isolation, yield, colony formation ability and differentiation capacity of USCs. The Matrigel culture group had two to three times more colonies after 5 days than the gelatine-coated Petri dish group. In addition, the number of cells cultured for 14 days after treatment with Y-27632 was increased by approximately 40-fold compared with that of cells cultured on gelatine-coated Petri dishes. Overall, Y-27632 and Matrigel provided the highest yield of isolated USCs (Kim et al., 2020a). When the cell fusion rate reached 80–90%, the cells were passaged at a ratio of 1:2 or 1:3 every 2–3 days. Fourth and tenth generation cells were reported to exhibit a pebble morphology (Wu et al., 2018), while other studies described them as stone-like (Kang et al., 2015) or grain-like (Lang et al., 2013; Zhang et al., 2021). However, to produce a large number of differentiated cells before transplantation, maximum expansion of USCs is often required *in vitro*, which results in cell ageing after repeated passages (Halley et al., 1988). Kang et al. seeded USCs at a density of 500 cells/cm^2^ in 24-well plates and found that their growth ability decreased gradually as the number of passages increased (Kang et al., 2015). Liu et al. mixed nondifferentiated USCs used at an early *in vitro* passage (≤*p3*) with an hp-HA gel containing growth factors, and the cells exhibited an improved viability, engraftment ability and vascular, neural and myogenic differentiation potential after subcutaneous implantation *in vivo* (Liu et al., 2020a).

## Underlying Mechanisms of USCs

### Regulating Immune Systems

**FIGURE 1:**
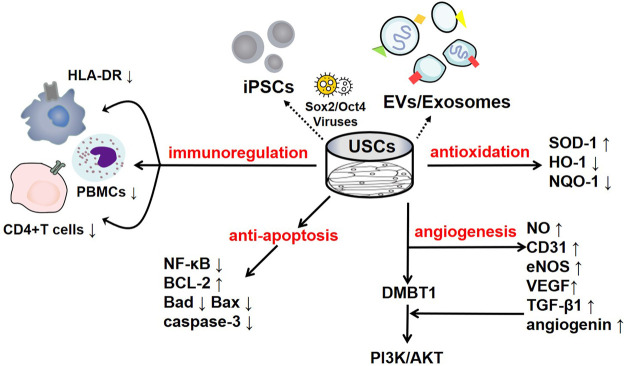
The regulatory mechanisms of USCs and their secreted products. USCs and their secreted products, such as exosomes, extracellular vesicles, and USC-iPSCs, play an important role in disease the pathogeneses. USCs exert negative immunoregulatory functions and serve as antioxidants, as manifested by their increased SOD-1 levels and reduced HO-1 levels. USCs exert an antiautophagic effect, characterized by increased p62 levels and decreased levels of LC3-II and Beclin1. Furthermore, USCs promote angiogenesis mediated by VEGF, angiogenin, and PI3K/AKT pathway activation. Interestingly, their effect on apoptosis remains controversial, as they exhibit increased levels of the apoptotic protein Bax and decreased levels of the antiapoptotic protein Bcl-2.

To date, some studies have found that USCs can affect immune system regulation. Wu et al. found that the lack of costimulatory molecules and MHC-II markers on cultured USCs indicates that they can escape recognition by the recipient’s immune system due to their low immunogenicity. In addition, the coculture of USCs with human peripheral blood mononuclear cells (PBMCs) significantly increases the USC expression levels of immunomodulatory cytokines (such as IL-6 and IL-8) and immunomodulatory chemokines (such as MCP-1). The secretion of cytokines (such as RANTES, GRO-α and GM-CSF) indicates that USCs have good immunomodulatory properties (Wu et al., 2021). Zhou et al. cocultured USCs with human CD4^+^ T cells stimulated with phytohemagglutinin (PHA) and observed that CD4^+^ T cell proliferation was inhibited in a dose-dependent manner and that the mRNA levels of IFN-γ, T-bet, IL-17A and RORC were significantly reduced. USCs can regulate CD4^+^ T cell activation and the Th1/Th17 immune response (Zhou et al., 2020). In addition, USCs reduce myeloperoxidase (MPO) activity and the number of MPO+ cells in the tissue, indicating reduced neutrophil infiltration. Therefore, in a mouse model of inflammatory bowel disease induced by dextran sulfate sodium or 2,4,6-trinitrobenzene sulfonic acid, the expression of proinflammatory factors was significantly reduced after treatment with USCs, thereby reducing the inflammatory response and improving the survival rate of the mice (Zhou et al., 2020). Similarly, Kang et al. cocultured USCs with PBMCs and observed a dose-dependent inhibitory effect on PBMC proliferation induced by USCs. Lymphocyte proliferation was further inhibited as the number of USCs increased (Kang et al., 2015). Additionally, Choi et al. found a low expression level of HLA (human leukocyte antigen)-DR in USCs, indicating their suppressed immunogenicity (Choi et al., 2017). HLA-DR is a surface receptor on MHC class II cells that plays an important role in antigen recognition, presentation, immune response and regulation. The absence of class II HLA expression enables the allograft to escape the recognition of antigen-presenting immune system cells; thus, there is basically no risk of immune rejection, and severe complications such as transplantation failure, late rejection and graft-versus-host-disease (GVHD) are reduced (Choi et al., 2017; Kot et al., 2019). All of the results of the studies mentioned above suggest that USCs negatively regulate immunoreactions and that this immunomodulatory effect is an efficient way to reduce inflammation. The various underlying mechanisms are summarized in Figure 1.

### Modulating Oxidative Stress

Oxidative stress is a harmful process that can lead to a variety of diseases, including cancer, cardiovascular diseases, neurological disorders, respiratory diseases, kidney diseases, chronic inflammation and accelerated ageing (Pizzino et al., 2017). Upon stimulation, the body produces many free radicals, such as reactive oxygen species (ROS) and reactive nitrogen species (RNS), which disrupt oxidation homeostasis and antioxidant mechanisms, leading to oxidative stress (Pizzino et al., 2017). The effect of USCs on the regulation of oxidative stress in the body is highly significant. Superoxide dismutase (SOD-1), which can inhibit tissue damage caused by free radicals, is considered a key antioxidant in the assessment of mitochondrial oxidative activities, and malondialdehyde (MDA) is considered to be an important marker of oxidative stress (Marrocco et al., 2017). Li et al. found that USC-derived exosomes (USC-Exos) reduced oxidative stress by increasing the activity of SOD and reducing the MDA content, thereby protecting human kidney cortex/proximal tubule cells (HK2) from hypoxia-reoxygenation (H/R)-induced damage (Li et al., 2020). Xiong et al. reported that treatment with USCs in a diabetic nephropathy (DN) model slowed the progression of renal fibrosis due to the obvious downregulation of nicotinamide adenine dinucleotide phosphate (NADPH) oxidase levels, whose increase promotes the progression of DN (Xiong et al., 2020). Similarly, Zhang et al. found elevated levels of SOD-1 in USC-treated rats with chronic kidney disease (CKD) (Zhang et al., 2020a). Moreover, the antioxidant effect of USCs has been observed in interstitial cystitis, which manifests as notably decreased levels of oxidative stress-associated indicators, including haem oxygenase (HO)-1 and NADPH quinine oxidoreductase (NQO)-1 (Li et al., 2017). Previous studies have demonstrated that USCs can alleviate oxidative stress damage in cells and have a certain therapeutic effect on kidney diseases.

### Enhancing Angiogenic Activities

Angiogenesis is a critical process involving the repair and regeneration of tissues exposed to severe ischaemia/hypoxia (such as wound healing, myocardial ischaemia, cerebral ischaemia, and cancer) and is mediated by endothelial cell (EC) proliferation and migration (Kargozar et al., 2020). As the time prior to the reception of revascularization therapy increases, the risk of endothelial dysfunction is markedly elevated. Hence, clinical investigations of rapid revascularization represent an excellent approach to improve patient prognosis.

As an endothelial marker, CD31 has been widely used to evaluate the density of blood vessels. Zhao et al. discovered dramatically elevated CD31 levels in the islets of mice treated with low-dose streptozotocin (MLD-STZ) after USC transplantation, indicating that USCs can help rebuild the islet architecture (Zhao et al., 2018). Additionally, proangiogenic microRNAs provided by USCs, such as miR-10, miR-21, and miR-30, have been found to participate in angiogenic growth in rats with diabetic erectile dysfunction (DED), as shown by the enhanced expression of CD31 and endothelial nitric oxide synthase (eNOS) (Ouyang et al., 2019). In USC-polycaprolactone/gelatine-treated wounds, the microvessel density was increased compared with that in the controls, and this stimulatory effect was prompted by vascular endothelial growth factor (VEGF) and TGF-β1, providing compelling evidence that the paracrine factors released by USCs play a key role in angiogenesis (Fu et al., 2014). Jiang et al. concluded that USC-Exos could promote angiogenesis by increasing the expression levels of VEGF, TGF-β1, and angiogenin (Jiang et al., 2016). Similarly, the deleted in malignant brain tumour 1 (DMBT1) protein is enriched in USC-Exos, and DMBT1 has been proven to function as a promoter of angiogenesis that activates the PI3K/AKT pathway by increasing the secretion of VEGF, nitric oxide (NO) and angiogenin (Karar and Maity, 2011; Chen et al., 2018a). Chen et al. showed that when human microvascular endothelial cells were exposed to USC-Exos, the proliferation of wound healing-related cells, such as fibroblasts and masses of capillary-like structures, was activated; when DMBT1 expression was suppressed, the functions of the USC-Exos were remarkably inhibited, as manifested by low levels of VEGF-A and Akt phosphorylation (Chen et al., 2018a). Thus, USCs clearly play a role in angiogenesis.

### Regulating Apoptosis

Apoptosis is a programmed cell death process that involves the formation of distinct apoptotic bodies to maintain homeostasis and regulate the regular cell cycle. However, excessive apoptosis can further impair tissue function, which, in turn, leads to more severe pathological conditions (Xu et al., 2019). Therefore, inhibiting apoptosis is regarded as rescuing impaired tissue. Several signalling pathways, such as the NF-κB and Bax/Bcl-2 pathways, and apoptosis-associated proteins, such as cleaved caspase-3 and metalloproteinase 1 (TIMP1), are involved.

The Bcl-2/Bax pathway is an important component of the endogenous apoptosis pathway. After the apoptosis pathway is activated, cells express the proapoptotic protein Bax to promote the release of cytochrome C (Cyt C) into the cytoplasm, which initiates the caspase cascade and leads to apoptosis. Sun et al. emphasized that USCs downregulated the expression of the proapoptotic proteins Bax and Caspase 3 and upregulated the expression of the antiapoptotic protein Bcl-2 in acute kidney injury (AKI) models. Compared with those in the control group, the renal function and histological damage of NRK-52E cells induced by cisplatin were significantly improved, and the proliferation of renal tubular epithelial cells was significantly increased (Sun et al., 2019). Similarly, in an interstitial cystitis model, treatment with USCs downregulated apoptotic levels by markedly reducing the levels of caspase-3 and Bax and notably increasing the level of Bcl-2, and fewer apoptotic nuclei were observed (Li et al., 2017). Moreover, USCs combined with chondroitinase ABC (chABC) can suppress neuronal apoptotic levels with high Bax and low Bcl-2 levels when administered to rats with spinal cord injuries (Li and Wu, 2017). ChABC functions by degrading the glycosaminoglycan side chains of chondroitin sulfate proteoglycans, ameliorating the inhibition of glial scar regeneration.

USCs and their secreted products are involved in the regulation of apoptosis. Jiang et al. found that after exposure to high glucose, podocytes and tubular epithelial cells of diabetic rats become damaged and overexpress caspase-3; nevertheless, USC-Exos protect podocytes and renal tubular epithelial cells by downregulating Caspase-3 overexpression and inhibiting apoptosis (Jiang et al., 2016). More importantly, these authors confirmed the role of USC-Exos in ameliorating human podocyte apoptosis in their follow-up experiment (Xiong et al., 2020). TIMP1 is a natural inhibitor of matrix metalloproteinases (MMPs). The recombinant human TIMP1 protein inhibited apoptosis by activating the PI3K and JNK signalling pathways. Chen et al. found that TIMP1 was highly enriched in USC-EVs and could enhance the antiapoptotic effects of OBs and endothelial cells and thus play a protective role (Guo et al., 2006; Chen et al., 2020). In addition, miR-146a-5p delivered by USC-Exos was reported to inhibit the expression of interleukin-1 receptor-associated kinase 1 (IRAK1) *via* posttranslational expression and significantly reduce NF-κB P65 nuclear localization, thus inhibiting the IRAK1-NF-κB signalling pathway (Li et al., 2020). Similarly, abundant miR-216a-5p carried in USC-Exos was shown to be transferred to human proximal tubular epithelial cells (HK-2) and further promote phosphatase and tensin homolog (PTEN) silencing and the activation of Akt phosphorylation, thereby protecting HK-2 cells from apoptosis (Zhang et al., 2020b). The various underlying mechanisms are summarized in Figure 1.

## Applications of USCs

### Renal Diseases

#### Chronic Kidney Disease

Renal diseases and complications are regarded as important factors leading to high hospitalization rates and a poor quality of life and pose substantial burdens on patients. The global prevalence of CKD ranges from 11 to 13%, and the global mortality rate represents 4.6% of the total mortality rate; CKD was the 12th leading cause of death in 2017 (Hill et al., 2016; GBD Chronic Kidney Disease Collaboration, 2020). As kidney tissue-specific stem cells, USCs may be the best choice for kidney tissue repair and have promise as a treatment for CKD. Zhang et al. found that the implantation of USCs protected the number of nephrons and significantly improved renal function in a rat CKD model due to their strong proliferation ability and ability to reduce fibrin matrix formation and inhibit renal inflammation. Therefore, USCs may improve renal function and prevent tissue structure damage caused by CKD in the clinic (Zhang et al., 2020a).

#### Renal Regeneration

Due to the high cost of haemodialysis and the shortage of kidney sources, renal regeneration is considered a possible and effective means to treat renal failure. However, the kidney is one of the most difficult organs to regenerate, and previous stem cells strategies have failed. Therefore, because they are derived from the kidney, readily available and safe, researchers have attempted to determine whether USCs can promote kidney tissue regeneration.

Therefore, we have confirmed that USCs play an active role in the repair of kidney tissue, but doubts remain about their ability to regenerate kidney tissue. After using human kidney differentiation medium for 2 weeks, Choi and others observed decreased expression of stem cell markers (SSEA4) and increased expression of renal lineage-specific markers (PAX2, WT1 and Cadherin-6), suggesting that USCs have a significant renal cell-lineage differentiation ability. In addition, USCs can secrete growth factors such as VEGF and PDGF-BB and do not induce histological abnormalities, suggesting their safety *in vivo*. Therefore, USCs may be a promising treatment for renal regeneration due to their good renal spectrum differentiation ability, accessibility and enhanced safety. However, due to the limitations of *in vitro* experiments, no kidney tissue-like morphologies have been observed and need to be confirmed by subsequent *in vivo* experiments, which will be the focus of future research. Thus, to date, the utility of USCs in renal tissue regeneration has not been demonstrated (Choi et al., 2017).

#### Acute Kidney Injury

AKI is an important clinical disease characterized by tubular injury and a rapid decline in renal function. At present, there is no method for complete recovery (Guo et al., 2019). AKI occurs in approximately 20% of hospitalized patients, and the mortality rate can reach 50% without renal transplantation (Levey and James, 2017). Therefore, alternative therapies are the key to improving the treatment and prognosis of patients with AKI. Studies have shown that USCs can inhibit inflammation in the kidney and significantly improve renal function and histological damage, suggesting their potential in the treatment of AKI and representing a new clinical treatment strategy. Tian et al. created rat models of ischaemic AKI and found upregulated expression levels of anti-inflammatory factors (IL-10 and TGF-β1) and downregulated levels of proinflammatory factors (INF-γ and IL-1β) in the USC-treated group, indicating that USCs exerted a good anti-inflammatory effect (Tian et al., 2017). Sun and others concluded that USCs alleviated renal function damage in a cisplatin-induced AKI model, which was manifested by reduction in blood urea nitrogen (BUN) and serum creatinine (SCr) levels and improvements in histology and ultrastructure. *In vitro*, the authors cocultured cisplatin-induced NRK-52E cells with USCs, and the cells exhibited a higher viability and lower apoptosis rate than the control group cells (Sun et al., 2019).

#### Diabetic Nephropathy

DN has emerged as the main cause of end-stage renal disease, especially since the number of DN patients is expected to reach 3 billion by 2025 (Xiong et al., 2020). Studies have shown that USCs can protect podocytes in patients with DN and inhibit apoptosis while improving renal tissue fibrosis. Jiang et al. investigated STZ-induced rat models and found marked increases in their urine volume and urinary albumin concentration, alleviated mesangial expansion and downregulated expression of podocyte survival factors, such as bone morphogenetic protein 7 (BMP-7). On the other hand, these effects were markedly attenuated after treatment with USC-Exos, indicating that USC-Exos are highly important for the survival of podocytes and endothelial cells (Jiang et al., 2016). Subsequently, Duan et al. proposed that USC-Exos can secrete microRNA-16-5p to inhibit podocyte apoptosis by suppressing high glucose-induced VEGF-A expression, as demonstrated by the upregulation of the podocyte surface marker protein nephrin and downregulation of glomerular smooth muscle-specific proteins (α-SMA) and apoptotic proteins (Bax and Caspase-3). Furthermore, microRNA-16-5p was overexpressed following an intravenous injection of USC-Exos and may have protected podocytes in a diabetes rat model (Duan et al., 2019). Moreover, Xiong et al. observed that USCs had renoprotective effects, as they protected against renal interstitial fibrosis, as demonstrated by remarkable reductions in BUN and SCr levels, improved fibrous hyperplasia in renal tissues and reduced expression of α-SMA (Xiong et al., 2020).

### Bladder Diseases

#### Bladder Reconstruction

Infection, cancer, trauma, inflammation, and iatrogenic injury can all cause bladder dysfunction, resulting in a reduced ability to effectively store and empty urine. The best method of urethral reconstruction after radical cystectomy is ileal cystoplasty or neobladder (Serrano-Aroca et al., 2018). However, bladder reconstruction using gastrointestinal tissue and other materials produce a series of sequelae and complications, such as metabolic abnormalities and chronic urinary tract infection, which substantially affect patient quality of life; thus, bladder reconstruction remains difficult. Therefore, tissue engineering has potential as an alternative method for bladder reconstruction, but it is not currently available in clinical practice and requires further study.

Utilizing the properties of basic fibroblast growth factor (bFGF) (providing a favourable microenvironment for the proliferation of mesenchymal cells and the production of ECM), Lee et al. studied a partial cystectomy rat model and found a synergistic effect between USCs and heparin-immobilized bFGF-loaded scaffolds, as demonstrated by an elevated bladder capacity, compliance or decreased inflammation and tissue regeneration. These authors proposed that this novel composite biomaterial represents a promising therapeutic strategy for bladder reconstruction and provides the possibility for the structural and functional repair of damaged tissues; however, problems, such as vascularization and neurotization, remain and must be solved before clinical application (Lee et al., 2015). Li et al. conducted an experiment on female rats with protamine/lipopolysaccharide-induced interstitial cystitis and found that USCs had a restorative effect on bladder function reconstruction by inhibiting oxidative stress, inflammatory reactions, and apoptosis (Li et al., 2017). Inflammation-related factors, such as lower IL-6 and TNFα levels and reduced mast cell infiltration, were observed in the urinary bladders of the IC control group but were markedly improved in the IC + USC groups. Although induced rat models cannot completely replicate diseases in humans, such as interstitial cystitis, these findings provide a solid basis for a prospective clinical trial.

#### Overactive and Underactive Bladder

Overactive bladder (OAB), manifesting as urinary urgency and frequent nocturia, is often treated with invasive botulinum toxin or neuromodulation and reconstructive surgery (Robinson and Cardozo, 2019). Large conductance voltage and Ca2+-activated K+ (BK) channels have been noted as excellent therapeutic candidates for OAB based on their effectiveness at reducing sexual excitability and the contractility of bladder smooth muscle by hyperpolarizing the membrane (Li et al., 2019). Interestingly, stem cells have multifarious ion channels that are thought to be involved in cell proliferation. Wang et al. proposed that the overexpression of BK channels in USCs can modulate cell growth (G1/S phase) and apoptosis. When these authors applied the BK channel antagonist iberiotoxin, they found elevated apoptosis rates in USCs, whereas decreased rates were observed after treatment with the BK agonist NS1619 (Wang et al., 2017b). Although these results are certainly exciting, the *in vitro* experiment did not necessarily reveal the same effect as the simulated experiment in the OAB models. Therefore, their study verifies only the effect of BK channels on USCs, and experimental data on the interactions of USCs are lacking. Whether USCs can serve as “vectors” to increase the effect of BK channels and therefore be useful as a treatment for OAB remains unknown.

Interestingly, the effect of USCs on underactive bladder (UAB) has been investigated. UAB is a complex urologic condition with severe complications, such as bladder outlet obstruction and stress urinary incontinence (SUI). Interstitial Cajal-like cells (ICC-LCs), pacemaker cells in the urinary system, have shown a downward trend in UAB (Feng et al., 2017) and have thus emerged as an ideal therapy to satisfactorily adjust spontaneous vesical contractions (Liu et al., 2017a; Ko et al., 2017). By transfecting lentiviral vectors with exogenous gene hyperpolarization-activated cyclic nucleotide-gated (HCN4) modification, Sun et al. inefficiently induced the directional differentiation of USCs into ICC-LCs, resulting in a morphological alteration from “rice grain”-like cells to spindle-shaped cells with multiple branches, higher expression of the ICC surface marker c-Kit, and a visible automatic depolarization current. Thus, the overexpression of HCN4 produced a preliminary ICC-LC-like phenotype in USCs. Additional studies could reveal the function of successful USC differentiation in the treatment of UAB (Sun et al., 2020).

### Urethral Diseases

#### Stress Urinary Incontinence

Urinary incontinence (UI) is the involuntary leakage of urine through the urethra. SUI, the most common type of UI, is caused by the involuntary loss of urine caused by dysfunction of the urethral sphincter or the pelvic floor muscles. The prevalence of SUI ranges from 10 to 40% in females aged greater than 50 years (Capobianco et al., 2018).

After the injection of a mixture of USCs, microbeads, and collagen gel-type 1 into mice, an improved myogenic differentiation capacity and enhanced revascularization, innervation, and tissue regeneration were observed, highlighting the possible utilization of this strategy in SUI (Liu et al., 2013). By establishing an SUI rat model *via* vaginal balloon inflation, Wu et al. found that USC-Exos play a role in treating SUI, as demonstrated by improved urodynamic parameters, recovery of pubococcygeal muscle tissue, and the activation of the proliferation and differentiation of muscle satellite cells by the phosphorylation of extracellular-regulated protein kinases (Wu et al., 2019). Therefore, USCs may be a new and feasible treatment for SUI, which is worthy of further exploration.

#### Urinary Tract Reconstruction

The toxic effects of urine can be isolated in urothelial cells, usually obtained through bladder biopsy, which can be invasive and damaging to the donor. However, contemporary studies have illustrated the potential role of the noninvasive and unlimited nature of USCs in noninvasive urinary tract reconstruction. Thus, USC-derived endothelial cells, urothelium and functional smooth muscle cells can be used to establish urinary tract mucosa, the urinary wall and blood vessels, respectively (Abbas et al., 2020).

Zhao et al. initially used a modified technique to prepare the vessel extracellular matrix (VECM), which comprises both the vascular basement membrane and the interstitial ECM and serves as a microporous seeding-cell structure, and found that VECM is likely involved in angiogenesis based on its traits, which are similar to those of VEGF. Then, these authors differentiated USCs into urothelial and functional contractile smooth muscle phenotypes using induction media containing factors such as TGF-β1 and miR-199a-5p. Finally, these authors combined these cells with VECM to successfully construct an intact and multilayered urothelium and lamellar dense connective smooth muscle after 2 months of ureter reconstruction in a rat urethral defect model, while the control group exhibited only one layer of discontinuous epithelial cells and a few smooth muscle bundles. If the tensile and rupture strengths of such regenerated ureters are satisfactory, tissue-engineered grafts might be applied for the treatment of long-segment ureteral defects in the future (Zhao et al., 2019). Liu et al. seeded USCs on the small intestinal submucosa (SIS) of a rabbit urethral defect model; compared with the control group rabbits (received only SIS and no USCs), these rabbits exhibited an ameliorated urethral calibre, an increased urothelial regeneration speed, a greater smooth muscle content, and a higher vessel density (Liu et al., 2017b). Wan et al. successfully induced the differentiation of USCs from six healthy adult individuals into urothelial cells, and the resulting cells were phenotypically comparable to the native urothelium and functionally comparable to their tight junctions based on their barrier function and ultrastructure (Wan et al., 2018). Analogously, Yang et al. differentiated rabbit USCs that were preexposed to PDGF-BB and TGF-β1 into not only smooth muscle cells expressing α-SMA but also urothelial cells expressing urothelial-specific proteins, including AE1/AE3 and E-cadherin, by exposing the cells to epidermal growth factor (EGF) (Yang et al., 2018). Although the authors of this paper did not conduct further experiments, there are reasons to believe that these two cell types that are efficiently differentiated by USCs may be used for lower urinary tract tissue regeneration.

### Diabetes

Diabetes is characterized by pancreatic β-cell destruction and insulin resistance and is well known as the ninth leading cause of death. Approximately 451 million people have been diagnosed with diabetes, and 5 million people died from its multiple comorbidities in 2018 or “and 5 million people have died from its multiple comorbidities since 2018 (Amanat et al., 2020; Lu and Zhao, 2020).” Although islet transplantation and replacement of pancreatic β-cells may serve as practical curative strategies, existing data support the applicability of USCs and as a new strategy for the treatment of diabetes.

Studies have confirmed that isolated USCs can differentiate into insulin-producing β cells (Hwang et al., 2019) and promote their survival and pancreatic islet angiogenesis (Zhao et al., 2018), which is suitable for the treatment of diabetes. USCs can be converted into insulin-producing cells, expressing insulin and glucagon mRNA and protein and secreting insulin in response to glucose stimulation. Due to their relatively more abundant source, rebuilding functional insulin-producing cells differentiated from USCs is better than performing organ grafting (Hwang et al., 2019). Accordingly, after USC transplantation resulted in islet vascular regeneration, improved glucose tolerance and islet morphology and enhanced insulin content were observed in MLD-STZ-treated mice, and it can be inferred that USCs may contribute to the survival of β cells by promoting the revascularization of islets. Moreover, improved blood glucose levels were observed in mice injected with USCs with high-dose STZ (Zhao et al., 2018). Unexpectedly, Dong et al. showed that the tail vein injections of USCs six times per week did not markedly reduce the fasting glucose levels in type II diabetic rats (Dong et al., 2016). Similarly, Ouyang et al. found that USCs had no significant effect on blood glucose after injecting USCs once into a sponge to investigate their effects on diabetic ED (Ouyang et al., 2014). Given that other studies investigating USCs showed improved glucose tolerance in diabetic animals, these results seem contradictory. Among the various approaches, intrapancreatic injections of USCs may be the most efficient approach, while intravenous injections have a limited effect on improving blood glucose because of the risk of trapping in the lung capillary (Dong et al., 2016). After some speculation, the conflicting data indicate that the dose and route of injection play a key role in treatment.

Complications of diabetes, including diabetic cardiomyopathy, kidney disease, neuropathy, retinopathy and bladder disease, are serious problems and can even be life-threatening (Cole and Florez, 2020). Because of their multidirectional differentiation and paracrine function, USCs are believed to be effective at reducing the loss of other tissues caused by diabetes. Dong et al. injected USCs into rats and found only small amounts of USCs in the pancreas and kidneys, while no USCs were observed in the heart and bladder. Additionally, these authors concluded that USCs could inhibit cell apoptosis and decrease the fibrosis index *via* a paracrine signalling mechanism, thereby improving left ventricular function and myocardial remodelling, rescuing glomerular function and increasing detrusor contractility (Dong et al., 2016). In summary, USCs may be a promising new and feasible strategy for the treatment of diabetes and its complications.

### Digestive System Diseases

Hepatocyte transplantation (HCT), a cell-based therapy, can be used to treat congenital metabolic disease and acute and chronic liver failure in patients lacking suitable donors (Anderson and Zarrinpar, 2018). Hu et al. found that a small number of USCs differentiated into hepatocytes after coincubation with hepatic progenitor cells, which indicated that USCs can be an alternative autologous stem cell source for HCT (Hu et al., 2020). Moreover, the transplantation of USCs into an acute liver injury model improved the levels of serum markers such as alanine aminotransferase (ALT) and aspartate aminotransferase (AST) and partially ameliorated pathological changes, indicating that USCs can partially restore liver function after acute liver injury (Hu et al., 2020). A hypoxic preconditioning strategy may influence the adaptation of transplanted cells *in vivo*. It has been reported that after hypoxic pretreatment, the liver recovery efficiency of USCs is slightly enhanced by the induction of autophagy in a chronic liver fibrosis mouse model (Hu et al., 2020). In addition, C-X-C motif chemokine (CXC) receptor 4 (CXCR4) was significantly upregulated in USCs after hypoxic preconditioning and then interacted with the (CXC) ligand 12 (CXCL12) (an important chemokine for cell transport and homing) expressed at high levels in damaged liver tissues, thus promoting the proliferation, colony formation and migration of USCs (Hu et al., 2021). Moreover, the cell fusion rate between USCs and hepatocytes was increased after hypoxic preconditioning, and these polyploid stem cells may be involved in liver regeneration (Hu et al., 2021). All of these findings confirm the value of hypoxic preconditioning in improving the therapeutic efficacy of USCs in patients with end-stage liver disease.

Although studies investigating USCs in the digestive system are limited, we can reasonably infer the mechanism based on existing research. As mentioned above, USCs can participate in negative immune regulation. It is well known that the gastrointestinal tract is the largest immune organ in the human body and contains numerous immune cells, gut microbes and their metabolites, which can alter immune homeostasis. In contrast, alteration of the intestinal flora can upset this balance and lead to a series of inflammatory reactions (Pickard et al., 2017). Thus, it is reasonable to assume that USCs may play a nonnegligible role the treatment of inflammatory bowel diseases, such as Crohn’s disease and ulcerative colitis. Moreover, USCs are highly significant in angiogenesis; thus, ischaemic bowel disease may be fundamentally curable by vascular remodelling based on intestinal wall ischaemia caused by mesenteric artery stenosis or occlusion mostly in elderly patients with atherosclerosis. More experiments are needed to test these hypotheses (Table 1).

**TABLE 1:** USC-based therapies for various diseases of bodily systems.

Disease		Model	Mechanism	Observations
Renal diseases	Chronic kidney disease	Chronic kidney disease (CKD) rat models	Antioxidative stress and antifibrotic activity	Reduced degrees of glomerular sclerosis and atrophic renal tubules, improved SCr and GFR Zhang et al. (2020a)
	Renal transplantation	USC	Decreased SSEA4 levels and gradual upregulation of kidney differentiation-related markers	Assessment of renal cell-lineage differentiation ability Choi et al. (2017)
	Acute kidney injury	Rat models of ischaemic AKI	USC-based treatment	Upregulated levels of interleukin-10 and TGF-β1, downregulated levels of interferon-γ and IL-1β Tian et al. (2017)
		Models of cisplatin-induced AKI	USC treatment *in vivo*; coculture of cisplatin-induced NRK-52E cells with USCs *in vitro*	Reduced BUN and SCr levels; higher cell viability and a lower apoptosis *in vitro* Sun et al. (2019)
	Diabetic nephropathy	STZ-induced rat models	USC-Exo treatment	Increased urine volume and albumin, downregulation of the podocyte survival factor BMP-7 Jiang et al. (2016)
		Podocytes treated with USC-Exos	Synergistic effect of USC-Exos and microRNA-16-5p	Protects podocytes *via* VEGFA Duan et al. (2019)
		Renal tissues	USC-targeted treatment	Reduced levels of BUN and SCr, improved fibrous hyperplasia, reduced expression of α-SMA Xiong et al. (2020)
Bladder diseases	Bladder reconstruction	Partial cystectomy rat model	Heparin-immobilized basic fibroblast growth factor-loaded scaffolds	Elevated bladder capacity, compliance or decreased inflammation and tissue regeneration Lee et al. (2015)
	Overactive bladder	Large conductance voltage and Ca2+-activated K+ (BK) channels in USCs	Overexpression of BK channels in USCs	The BK channel antagonist iberiotoxin increased the apoptosis of USCs; USC apoptosis was decreased by treatment with the BK agonist NS1619 Wang et al. (2017b)
	Underactive bladder	Preliminary ICC-LC-like phenotype of USCs	Differentiation of USCs into ICC-LCs by the transfection of lentiviral vectors with exogenous gene modifications	Higher c-Kit expression, an automatic depolarization current Sun et al. (2020)
Urethral diseases	Stress urinary incontinence	Mice	Injection of USCs, microbeads and the collagen gel-type 1	Improved myogenic differentiation, enhanced revascularization and innervation, tissue regeneration Liu et al. (2013)
		SUI rat models	Treatment of USC-Exos with phosphorylated extracellular-regulated protein kinases	Improved urodynamic parameters, recovered pubococcygeus muscle tissue Wu et al. (2019)
	Urinary tract reconstruction	USCs; tissue-engineered grafts	Induction of media with components such as TGF-β1 and miR-199a-5p	Differentiation of USCs into urothelial and functional contractile smooth muscles Zhao et al. (2019)
		Urethral defect rabbit models	Seeding of USCs onto the small intestinal submucosa	Ameliorated urethral calibres, sped up urothelial regeneration, increased smooth muscle content Liu et al. (2017b)
		USCs from healthy adults	Induction of USC differentiation into urothelial cells	Structures phenotypically and functionally comparable to those of the native urothelium Wan et al. (2018)
		USCs from rabbits	Exposure to PDGF-BB and TGF-β1	High expression of α-SMA and urothelial-specific proteins (AE1/AE3 and E-cadherin) Yang et al. (2018)
Diabetes		USCs	Conversion to insulin-producing cells	High mRNA levels of the pancreatic transcription factors Pdx1, insulin and glucagon Hwang et al. (2019)
		Transplanted USCs from mice that were injected with high-dose STZ	USC transplantation to promote islet vascular regeneration	Improved glucose tolerance and islet morphology, enhanced insulin content, improved blood glucose Zhao et al. (2018)
		Type II diabetic rats	Tail vein injection of USCs six times every week	Did not markedly reduce fasting glucose levels Dong et al. (2016)
		Mice	Single injection of USCs into a sponge	Had no significant effect on blood glucose Ouyang et al. (2014)
Digestive system diseases	Hepatocyte transplantation	Chronic liver fibrosis mouse model	Promotion of autophagy, proliferation, colony formation, migration and cell fusion	Enhanced liver recovery efficiency Hu et al. (2020), Hu et al. (2021)
Nervous system diseases	Neurogenesis	Mouse brain	Seeding of USCs onto a hydrogel scaffold and transplantation into the rat brain	Survived at the lesion site with a great growth rate, differentiated into neuron-like cells Guan et al. (2014)
		USCs	Combination of laminin and platelet-derived growth factor-BB	Increased levels of neuronal markers (MAP2, NFM and NeuN) Kim et al. (2018)
		USCs in chemical-only induction protocol	Induction of ISX9, I-BET and RA; improved conversion of USCs into neuronal cells	Increased levels of neuron-specific markers (Tuj1, Map2 and Tau), improvement of electrophysiological properties Liu et al. (2020b)
	Spinal cord injury	Spinal cord injury rat models	Elevated expression levels of nerve growth factors and brain-derived neurotrophic factors	Improved motor function in rats Li and Wu (2017), Chen et al. (2018b)
	Ischaemic stroke	Rat models of ischaemic stroke	USC‐Exo injection; increased number of EdU+/Nestin+ cells in the subventricular zone	Attenuated neurological deficits, reduced infarct volume Ling et al. (2020)
		Oxygen‐glucose deprivation/reoxygenation-processed NSCs	USC‐Exos; exosomal microRNA‐26a	Exerted neurogenic effects on the suppression of histone deacetylase 6 (HDAC6) Ling et al. (2020)
Locomotor system diseases	Osteoporosis	Ovariectomized rat models	USC-EVs; mediated by the collagen triple-helix repeat containing 1 (CTHRC1) and osteoprotegerin (OPG) proteins	Increased bone mass, effective for the treatment of osteoporosis Chen et al. (2019)
			Treatment of USC-EVs to promote osteoblastic bone formation	High levels of osteoblast formation-related mRNAs (osteocalcin, Alp, and Runx2) Chen et al. (2019)
	Muscle regeneration	Mice	USCs promote skeletal muscle regeneration	Specific skeletal muscle lineage cell transcripts and protein markers such as myf5, myoD and myosin Chen et al. (2017)
		USCs	Combination of USCs and growth factors; hyaluronic-heparin hydrogel scaffold	Increased muscular cell survival rate Liu et al. (2020a)
		Mice with hindlimb suffering due to ischaemia	Transplantation of USC-EVs; angiogenesis	HMEC-1 and C2C12 cell proliferation, muscle regeneration Zhu et al. (2018)
Cutaneous regeneration and wound healing		Rabbit full-thickness skin defect models	Uses of biocompatible polycaprolactone/gelatine nanofibrous membrane scaffolds	Improved wound contraction, skin appendage regeneration, reepithelialization and neovascularization Fu et al. (2014)
		Human umbilical vein endothelial cells (HUVECs)		Significantly enhanced the proliferation, motility and tube formation ability of HUVECs Fu et al. (2014)
		Endothelial cells, full-thickness excisional wounds	Paracrine effects	Improved the proliferation of endothelial cells, promoted fibroblast differentiation, increased the levels of vWF, collagen and fibronectin Zhang et al. (2018)
		Rat full-thickness skin wound models	Proliferation and survival of EAhy926 cells	Accelerated collagen deposition and angiogenesis Cao et al. (2019)
			Seeding of USCs onto a small SIS scaffold in preconditioned hypoxia	Increased the secretion of VEGF, collagen and elastic fibre s Zhang et al. (2020c)
		Streptozotocin-induced diabetic mice	High expression of DMBT1	Sped up revascularization and collagen deposition Chen et al. (2018a)
Periodontal tissue engineering		Human periodontal ligament stem cells (PDLSCs)	Noncontact coculture of USCs; improved proliferation and osteoblastic/cementoblastic differentiation of PDLSCs	Increased the density of collagen layers, the levels of the cementogenic protein and ALP activity Yang et al. (2020)
			ECM derived from USCs	Enhanced proliferation, osteogenic differentiation potential, and angiogenesis Xiong et al. (2019b)
Erectile dysfunction		Bilateral cavernous nerve injury (CNI) rat models	Injection of USCs; lower rate of cell apoptosis	Markedly increased the ICP level and the ICP/MAP ratio, increased the ratio of smooth muscle to collagen in the corpus cavernosum Chen et al. (2018c)
			USCs modified with pigment epithelium-derived factor (PEDF); antiapoptosis	Exerted protective effects on nerves and ECs in subjects with erectile function Yang et al. (2016)
		Male rat models of streptozotocin injection	Treatment with USC-EVs	Increased endothelial expression and the smooth muscle content, increased the ICP level and the ICP/MAP ratio Ouyang et al. (2019)
		DED rats	USC-EVs; treatment	Increased endothelial expression and the smooth muscle content, increased the ICP level and the ICP/MAP ratio Zhou et al. (2019)

### Nervous System Diseases

Due to the absence of transplantable human neurons and insufficient technological means of neuron proliferation, challenges remain in the clinical treatment of neuronal apoptosis caused by spinal cord injury, acute brain ischaemia and neurodegenerative diseases (Fricker et al., 2018). The capacity of USCs to differentiate into neurons brings great hope for the revitalization of neurons, but increasing their proliferation and differentiation is urgently required. USCs have been proven to be important elements in the field of neuronal regenerative medicine. In addition, as a useful noninvasive cell source for disease modelling, USCs have high proliferation and differentiation capabilities and have great potential in neurological disease modelling (Shi and Cheung, 2021).

After seeding USCs onto hydrogel scaffolds and transplanting them into rat brains, Guan et al. found that USCs could not only survive at the lesion site with an excellent growth rate but also differentiate into neuron-like cells expressing neural phenotype-related proteins (Guan et al., 2014). In a study by Kim et al., the combination of laminin and platelet-derived growth factor-BB had a synergistic effect on the differentiation potential of USCs, as indicated by increased levels of neuronal markers, such as MAP2, NFM and NeuN (Kim et al., 2018). Additionally, Liu et al. used a chemical-only induction protocol involving the neuronal differentiation inducer ISX9, the disrupter of nonneuronal genes I-BET, and a metabolite of vitamin A1 (namely, RA) and observed that this treatment led to the improved conversion of USCs into neuronal cells based on the detection of the neuron-specific markers Tuj1, Map2 and Tau and their electrophysiological properties (Liu et al., 2020b). Clearly, USCs help with neurogenesis.

Regarding the therapeutic effects of USCs on diseases, Chen et al. transplanted USCs and chABC in combination into the impaired spinal cords of rats to evaluate functional injuries in their lower extremities, and both groups showed significantly improved motor function, which was attributed to the elevated expression of nerve GF and brain-derived neurotrophic factor (Li and Wu, 2017; Chen et al., 2018b). Notably, in rat models of ischaemic stroke, Ling et al. demonstrated that the injection of USC-Exos could attenuate neurological deficits and promote neuronal recovery, as indicated by a reduced infarct volume and enhanced neurogenesis. An increased number of EdU+/Nestin+ cells was observed in the subventricular zone, where adult neural stem cells (NSCs) mainly reside (Ling et al., 2020). In addition, using oxygen-glucose deprivation/reoxygenation-processed NSCs as *in vitro* models, these authors found that USC-Exos could transfer microRNA-26a to induce the degradation of histone deacetylase 6 (HDAC6) mRNA and inhibit the translation of HDAC6 mRNA, thereby increasing the self-renewal and differentiation of NSCs (Ling et al., 2020). The proneurogenic effects of USC-Exos *in vivo* may be related to the role of the miR-26a/HDAC6 axis, but further studies are needed for verification. In conclusion, USCs can enhance the proliferation and differentiation of neurons, providing a new idea for the treatment of diseases related to neuronal apoptosis.

### Locomotor System Diseases

#### Osteoporosis

Osteoporosis, characterized by a reduced bone mineral density and decreased bone strength, is a systemic bone disorder that predisposes patients to an increased risk of fracture; its pathogenesis is ascribed to the apoptosis of OBs and the proliferation of osteoclasts (OCs) (Miller, 2016). Because EVs secreted by USCs are capable of promoting healing in various damaged parenchymal tissues, researchers are attempting to use them to treat osteoporosis (Riazifar et al., 2017).

Chen et al. speculate that USC-EVs may promote bone formation and prevent the development of osteoporosis. To investigate this hypothesis, they used heterogeneous rat models of different ages, sexes and health conditions to confirm that USC-EVs express and mediate the collagen triple helium repeat containing 1 (CTHRC1) and bone protein (OPG) proteins and thereby promote bone formation and inhibit bone formation. How these proteins are sorted into USC-EVs and then reused by recipient cells remains unclear, and their effectiveness in preventing osteoporosis requires further investigation (Chen et al., 2019). The ability of USCs to proliferate and differentiate into OBs can be used for not only the treatment of osteoporosis but also the repair of large segmental bone defects (Liu et al., 2021). In addition to their remarkable osteogenic ability, USCs have a cartilage repair effect similar to that of BMSCs *in vivo* and can be used as a stem cell replacement for cartilage regeneration, providing a powerful platform for clinical transformation (Sun et al., 2021). Some researchers have pointed out that USCs may serve as a new treatment option for osteoporosis.

#### Muscle Regeneration

Skeletal muscle is one of the largest organs in the human body. Healthy and complete skeletal muscle has decisive effects on human health and quality of life. Muscle injury and regeneration imbalance lead to the occurrence and development of diseases, and while muscle satellite stem cells (MuSCs) are the main source of muscle regeneration, their function gradually decreases over time. Therefore, alternative therapies are urgently needed (Yamakawa et al., 2020). USCs have the potential for myoblast differentiation and may be significant candidates for skeletal muscle regeneration, as indicated by their expression of specific skeletal muscle lineage transcripts and protein markers, such as myf5, myoD, and myosin (Chen et al., 2017).

After the implantation of USCs cultured in myogenic differentiation medium into the anterior tibialis muscles of mice, skeletal muscle lineage cell markers were found to be stably expressed, suggesting that USCs play a role in skeletal muscle regeneration (Chen et al., 2017). Since long-term muscular conversion from USCs *in vitro* always leads to high cell death and low differentiation, researchers have explored novel methods to improve their survival rate *in vivo* by applying primary USCs to a hyaluronic-heparin hydrogel containing various GFs, such as myogenic-related IGF1, HGF, and PDGF-BB. The increased cell survival rate and the expression of myogenic and endothelial cell markers revealed the combinatory effect of USCs and GFs on muscle tissue engineering (Liu et al., 2020a). After transplanting USC-EVs into mouse hindlimbs suffering from ischaemia, remarkably higher levels of angiogenesis, HMEC-1 and C2C12 cell proliferation, and muscle regeneration were observed by Zhu et al. (2018). Thus, their achievement suggests a novel therapy for attenuating hindlimb ischaemic injury in the future.

### Other Diseases

#### Cutaneous Regeneration and Wound Healing

Appropriate skin tissue scaffolds can promote wound healing, but the lack of appropriate scaffold materials is an obstacle in skin tissue engineering. The available data have shown that USCs combined with some novel biomaterials can promote neovascularization, which is a crucial part of the wound healing process. As a result, USCs, which have multidirectional differentiation potential, have become an important strategy for the construction of engineered skin tissue to promote the healing of extensive skin injuries caused by wounds, ulcers, burns and inflammation.

Fu and coworkers fabricated biocompatible polycaprolactone/gelatine nanofibrous membranes as scaffolds for wound repair and showed that they had a mechanical strength similar to that of human skin tissue; they then seeded USCs onto these composite meshes. Compared to that in rabbit full-thickness skin defect models that were treated with the PCL/GT membrane alone, markedly accelerated wound healing was observed following USC-PCL/GT treatment, which manifested as remarkably improved wound contraction, thicker granulation tissue, regeneration of skin appendages (such as hair follicles), faster reepithelialization and neovascularization, higher levels of collagen and higher capillary density (Fu et al., 2014). In an *in vitro* experiment, after culturing human umbilical vein endothelial cells (HUVECs) in USC-conditioned medium, the authors observed significantly enhanced proliferation, motility and tube formation abilities of the HUVECs (Fu et al., 2014). Bioglass, which is a type of bioactive silicate, is a special glass that is directly connected to living bone or soft tissue to repair damage. Bioglass can upregulate the secretion of VEGF and bFGF from USCs; this product is called bioglass-activated USCs and can enhance the wound healing ability of endothelial cells and fibroblasts. Using this property, Zhang et al. confirmed that USCs combined with bioglass could promote wound healing by activating the paracrine effects between USCs and recipient cells by applying bioglass-activated USCs to full-thickness wounds. This effect was manifested as improvements in endothelial cell proliferation and myofibroblast differentiation into fibroblasts, upregulated VEGF and bFGF levels, capillary-like network formation and elevated levels of vWF, collagen and fibronectin (Zhang et al., 2018). By combining USCs with biomaterials, namely, surface-structured bacterial cellulose (S-BC), Cao et al. found that USCs promoted the proliferation and survival of EAhy926 cells (a type of HUVEC) and that their synergistic effect accelerated collagen deposition and angiogenesis upon their evaluation of rat wound closure (Cao et al., 2019). Scholars have provided a novel idea, designated as hypoxic preconditioning, for the prevention and treatment of damage resulting from a hypoxic environment involving the repeated short-term exposure of tissues or cells to hypoxia; hypoxic preconditioning can improve tolerance to hypoxia. It is well known that this method improves the survival and proliferation of transplanted stem cells. Furthermore, in preconditioned hypoxia, USCs seeded on a SIS scaffold, which is a tool widely used in soft tissue repair, can increase the secretion of VEGF, bFGF, collagen, and elastic fibres to facilitate full-thickness skin wound healing in mouse models (Zhang et al., 2020c). Moreover, Chen et al. reported that USC-Exos have beneficial effects, such as accelerated revascularization and collagen deposition, on soft tissue wound repair in STZ-induced diabetic rats and that these effects may be attributed to the high expression of the proangiogenic protein DMBT1 (Chen et al., 2018a). USC-based skin stents may be a new method to promote skin wound healing, but they have not yet been used in clinical treatment.

#### Periodontal Tissue Engineering

Human periodontal ligament stem cells (PDLSCs) are considered to be an important material for periodontal tissue regeneration and can be used in the treatment of diseases such as periodontitis and gingivitis. PDLSCs are largely not afflicted by the disadvantage of not allowing complete periodontal tissue regeneration affecting current clinical treatment strategies. However, due to the limited number of PDLSCs, developing strategies to promote cell proliferation and differentiation is currently the top priority.

There is evidence of improved proliferation and osteoblastic/cementoblastic differentiation in PDLSCs, as demonstrated by denser collagen layers and elevated levels of the cementogenic protein cementum protein 1 (CEMP1), alkaline phosphatase (ALP) activity, runt-related transcription factor 2 (RUNX2) and osteocalcin (OCN) in a noncontact coculture of USCs (Yang et al., 2020). Additionally, Xiong et al. found that ECM derived from USCs enhanced the proliferation, osteogenic differentiation potential, and angiogenesis of PDLSCs compared to native PDLSCs (Xiong et al., 2019b). These results indicate that USCs may be the most suitable substrate for the coacceleration of osteogenic reactions with PDLSCs, but they have not yet been applied in clinical practice.

#### Erectile Dysfunction

The average prevalence of ED, which is associated with many risk factors, such as diabetes, is 30% in patients of different ages. Due to their reduced quality of life, these patients often undergo high-cost penile prosthesis implantation, causing distressing complications, such as corporal fibrosis (Yafi et al., 2016). Many studies have shown that USCs play a key role in the treatment of ED.

USCs can improve erectile function and repair the endothelial structure of the cavernous body by protecting nerves, reducing fibrosis, and inhibiting cell apoptosis in cavernous tissue (Chen et al., 2018c). In the experiments performed by Yang et al., USCs modified with pigment epithelium-derived factor (PEDF) improved erectile function in rats with cavernous nerve injury (CNI) by exerting protective effects on nerves and ECs in combination with their antiapoptotic effects (Yang et al., 2016). According to the observations reported by Ouyang et al., USC-EVs ameliorate diabetic erectile dysfunction (DED) by increasing the endothelial expression and smooth muscle content in male rat models established by STZ injection (Ouyang et al., 2019). Similarly, Zhang et al. revealed that increased autophagy activity is related to the improved function of cavernous endothelial cells in the DED model, suggesting that the reversal of autophagy may serve as a new therapeutic target for DED (Zhou et al., 2019). However, the improvement of ED by USCs is based on animal experiments such as rats, and further clinical studies are needed to verify the results.

## Current Challenges

Despite the substantial potential of USCs, there are still challenges that need to be overcome. When the cells used in experiments are derived from individuals with different genetic backgrounds, their diversity can affect the experimental reproducibility (Ji et al., 2017a). The age, sex, and health status of the urine donor may affect the proliferation and pluripotency of stem cells; for example, USCs from the urine of young and healthy people have a higher ability to proliferate and differentiate into various lineages (Tayhan et al., 2017), while USCs from patients with diabetes have a significantly reduced ability to regenerate (Xiong et al., 2019a).

As mentioned above, USCs have multidirectional differentiation potential; however, given their decreased potential for osteogenic and chondrogenic differentiation compared with that of BMSCs, strategies to improve their directed differentiation efficiency are urgently needed (Ji et al., 2017a; Wu et al., 2018). Moreover, the differentiation of USCs has mostly been demonstrated *in vitro*, and their differentiation ability *in vivo* should be further explored. Preconditioning, including gene modification, drug pretreatment and coculture, can be used to enhance the differentiation ability of USCs *in vivo* (Hu et al., 2020). Additionally, Dong et al. suggested that USCs can differentiate into PDX1-positive cells in the rat pancreas (Dong et al., 2016). However, in 2018, Zhao et al. revealed that USCs could protect beta cells from apoptosis induced by minor-to-moderate injuries in STZ-induced diabetic mice; however, these cells could not replace the already dead beta cells (Zhao et al., 2018). These two contradictory results remain controversial and require further experimental evidence.

Before USCs can be formally applied in clinical practice, standardized products must be acquired and storage and transport, *in vitro* amplification, administration and dosing, and cell homing must be optimized. In addition, the curative effect of basic cell therapy must be improved, and the cell therapy protocol must be optimized.

Safety and effectiveness should also be considered. Recent research suggests that the proliferation and differentiation of implanted cells, such as USCs, and their corresponding scaffolds are in danger of reconstruction in the urinary tract due to the highly cytotoxic nature of urine (Abbas et al., 2020).

Reprogrammed iPSCs from USCs are affected by genomic instability and epigenetic memory associated with the reprogramming process, resulting in aberrant differentiation *in vitro* and even influencing disease modelling and clinical applications (Ji et al., 2017b). Teratoma formation is a major side effect of iPSC transplantation (Parrotta et al., 2019).

Exosomes derived from USCs represent a novel cell-free therapeutic strategy that have the advantages of long-term maintenance of biological activity, low immunogenicity and no promotion of tumour formation. Nevertheless, specifically labelling and tracking exosomes *in vivo* remains challenging (Li et al., 2020).

## Conclusion

Overall, USCs and their secreted factors play roles in immune regulation, oxidative stress modulation, revascularization, cell apoptosis and autophagy. Through the mechanisms above, USCs have substantial potential in tissue engineering and personalized therapeutic strategies and exert therapeutic effects on a variety of diseases, such as those of the urinary, nervous, locomotor, reproductive endocrine and digestive systems. Although several challenges currently exist, we anticipate that future research will identify solutions to the existing problems and expand the application range.
